# Knee Osteoarthritis in Patients With High BMI: The Role of an Orthopaedic Surgeon

**DOI:** 10.7759/cureus.48464

**Published:** 2023-11-07

**Authors:** Abdullah Hanoun, Ben Steele-Turner, Amit Chandratreya

**Affiliations:** 1 Trauma and Orthopaedics, Leeds General Infirmary, Leeds, GBR; 2 Physiotherapy, University of Portsmouth, Portsmouth, GBR; 3 Trauma and Orthopaedics, Princess of Wales Hospital, Bridgend, GBR

**Keywords:** weight loss, joint replacement, high bmi, obesity, osteoarthritis

## Abstract

Obesity and lower limb osteoarthritis (OA) are amongst the commonest conditions worldwide, with increasing burden on health systems. The relationship between the two is complex. Obesity is thought to be a risk factor for OA, and OA can hinder efforts to reduce weight. Total knee replacement (TKR) is a widely used and effective management for knee OA. However, high body mass index (BMI) can complicate the surgery, which leads to some surgeons denying this operation to patients above a certain BMI. Orthopaedic surgeons have an important part in helping patients lose weight in the process of preparing for their TKR. We review the effect of high BMI on developing symptomatic knee OA, the complication rate with high BMI and TKR and the obstacles to losing weight in the presence of OA and point to areas where the orthopaedic surgeon can find support for their patients during their journey to losing weight. We review the evidence to see whether denying patients a TKR based on their BMI is justified and look into the most effective way to engage high BMI patients to improve their chance of a complication-free TKR.

## Introduction and background

It is calculated that 1-2.5% of the gross national product of Western nations is used to meet the health costs of osteoarthritis (OA) [1]. The largest part is spent on knee and hip replacements [2]. Knee replacement is common in people with overweight or obesity [3,4]. 2.1 billion people worldwide are considered obese. Obesity prevalence and severity are rising globally [5].

The World Health Organization (WHO) defined Class III obesity, as a BMI > 40 kg/m^2^. This carries a significant health risk [6]. Obesity is a major concern for orthopaedic surgeons. High BMI is considered a risk factor for perioperative complications, in particular with total hip replacement (THR) or total knee replacement (TKR).

With the link between obesity and lower limb OA and total joint replacement, clinical guidelines for OA management around the world (including the recent UK’s National Institute for Clinical Excellence (NICE) Guideline update on OA management) recommend weight management as a core strategy for all OA patients [7].

One meta-analysis suggested that in order to have moderate pain relief for OA, weight loss of 5% within a 20-week period is needed [7,8]. With NICE guidelines recommending losing 10% weight before operations, the discussion regarding the importance of losing weight as part of managing OA would have taken place ideally before the referral to the orthopaedic clinic; however, the encounter is another chance to approach this subject which could be sensitive to some patients. Setting realistic targets and having a sensitive approach while being honest regarding the importance of the issue take a skill. A busy clinic rarely allows the subject to be covered fully. We aim to review the relationship between knee OA and obesity and review the pros and cons of operating on patients with high BMI. We specifically look at whether a BMI limit should be enforced before offering a TKR. We give pointers to the orthopaedic surgeon on how best to approach the subject with patients, within the limitation of a busy orthopaedic clinic, and groups that could offer support to the patient.

## Review

Association of high BMI and knee OA

To define the association between BMI and the risk of TKR, a total of 16,362 knees from 8,181 persons were studied [4]. There is a strong association between the change in BMI and the risk of knee replacement (adjusted hazard ratio 1.03; 95% confidence interval [CI] 1.00 to 1.06). Combining results from this study and three older studies [7,9,10], it seems that obesity has a strong association with an increased risk of TKR.

A dose-response relationship exists between symptomatic knee OA and BMI [11]. Leyland in a meta-analysis found that increasing the BMI by 5 translates to a 35% increased risk of knee OA [12].

In a secondary analysis of the Intensive Diet and Exercise for Arthritis (IDEA), (a randomised clinical trial, which grouped 240 overweight or obese symptomatic knee OA patients who successfully lost either; <5%, 5-10%, 10-20% or >20% of their initial body weight [13]), a significant dose-response relationship existed between percentage of body weight loss and improvements in health-related quality of life. This relationship was also shown by Riddle and Stratford who divided a cohort (n=1,410) of knee OA patients from the Osteoarthritis Initiative (OAI) and Multicentre Osteoarthritis (MOST) into five categories: ≥ 10% body weight loss, 5-9.9% loss, between 4.9% loss to 4.9% body weight gain, 5-9.9% gain and ≥ 10% body weight increase over a 30-month timeframe [14]. In this cohort, a significant dose-response relationship existed between WOMAC (Western Ontario and McMaster Universities Arthritis Index) Physical Function and WOMAC Pain with the degree of weight change in both directions. Those who lost ≥ 10% or gained ≥ 10% body weight saw a notable change in reported symptoms. A critique of this paper could be that those in the reference group varied between a 4.9% loss and 4.9% body weight gain. This is quite a wide variation in percentage weight change to be grouped as a reference. Nevertheless, it favours the ≥ 10% of body weight loss as an ideal target.

Joseph et al. followed 2,752 individuals over four years, tracking their BMI and clinical and radiological OA changes in their knees and hips [10]. Their findings suggest that weight changes affect radiographic and symptomatic knee OA. Four hundred and eighty-five participants were able to lose more than 5% of their original weight. However, 714 participants actually gained >5% of body weight during the study.

Outcomes of knee arthroplasty in patients with high BMI

Total joint arthroplasty is one of the most cost-effective procedures even in higher-risk patients [15]. Some research suggested high BMI to have a negative effect on the outcomes, which was contradicted by other research [16]. TKR can lead to either weight loss or gain depending on the study you read, with a substantial effect on post-operative outcomes [17]. It is, therefore, of particular importance to guide obese patients to realistic expectations in the pre-operative shared decision-making process [18].

Polat et al. concluded that for unicompartmental knee replacement, morbid obesity is an independent risk factor for functional outcomes and implant survival [19]. The 90-day costs were significantly greater in the morbidly obese and superobese relative to patients with smaller BMI, which was mainly due to readmissions. The change in outcome scores was similar across all the BMI cohorts for most of the patient outcome measures [20].

Does reducing BMI before TKR restore outcomes to the population level?

More evidence is needed to show whether losing weight is clinically significant and sufficient to improve outcomes after TKA [21]. Jin et al. found that (after adjusting for age, sex, BMI, socioeconomic and lifestyle factors) weight loss of >7.5% was associated with reduced risk of TKR (hazard ratio 0.69, 95%CI 0.54-0.87). This was not the case for THR [7].

Should we enforce a BMI threshold to offer a TKR?

Cleveland Clinic OME Arthroplasty Group concluded that setting a BMI threshold would deny surgery to patients who may benefit the most from it [22]. Specific BMI cut-offs are used by some hospitals when determining patients’ eligibility for surgery [23,24]. Some studies suggested that patients do gain weight post-THR [25], hence the suggestion of BMI cut-offs. Ast et al. found that THR had a positive effect on obese patients when it comes to losing weight [26].

Using 40 kg/m^2^ as a BMI cut-off, Giori et al. found that to prevent a single complica­tion, 14 patients had to be refused surgery [15]. These data suggest that enforcing strict BMI criteria may unjustifiably deprive the benefits of TJA for this large group of patients, especially since obese patients have been shown to have similar functional improvements after TJA compared to non-obese patients [27,28].

Is it possible to lose weight in the presence of lower limb OA?

In a recent study assessing patients’ demands prior to TKR, obese patients expect TKR as a means for weight reduction, through regaining the ability to exercise after surgery [18]. High BMI exacerbates knee OA, and in turn knee OA affects mobility and ability to exercise, which makes losing weight difficult for some patients. Those patients feel that the only way out is by having a joint replacement in the hope of breaking the cycle.

The IDEA trial recruited 454 high BMI patients with OA and grouped them into control, exercise, diet and exercise & diet intervention [29]. Three hundred and ninety-nine participants (88%) completed 18 months of the study. The diet group had lower knee compressive forces, and IL-6 levels were lower in diet and diet & exercise groups. More importantly, there was an average loss of weight of 1.8 Kg in the exercise group, compared to 8.9Kg for diet, and 10.6 Kg for the exercise & diet group, proving that patients who have high BMI and knee OA can lose weight through a combination of exercise and diet if the program was robust enough.

Furthermore, Peeler and Ripat were able to recruit 22 females with an average BMI of 34 and age of 63 years, into a 12-week program of activity consisting of a Lower Body Positive Pressure Treadmill program [30]. They were able to complete the program and they reported an improvement in Quadriceps strength, KOOS scoring and pain measurements. They all had either mild or moderate OA so the findings may not apply to advanced OA.

Joseph et al. showed that 17.6% of their participants were able to lose more than 5% of their original weight [10]. However, in the same paper, 25.9% actually gained >5% of body weight during the study.

What is the best target to set the patient when discussing weight loss?

Avery et al. studied a cohort (n=24,447) of participants who attended a commercial weight loss group and had a BMI of > 30kg/m^2^ [31]. They compared individuals who set a weight loss target versus those who had no specific target, finding those individuals who had a specific target were more likely to lose >10% of their body weight after 12 months than those without a set goal. They also found that those setting multiple incremental targets, and those with more ambitious targets had greater success at attaining clinically significant weight reduction.

A pivotal decision that must be made by orthopaedic surgeons recommending weight loss to a patient is determining the metric through which this recommendation is made. The blanket use of a BMI cut-off to determine suitability for TKR may not be well understood by some patients. However, weight loss should still be promoted by all clinicians to overweight or obese OA patients as a primary treatment [32-34].

This goal should be used to promote weight loss appropriately to each individual, whilst also suitable for surgeons to routinely recommend during busy clinics, and the advice should engage patients. It should have the patient’s overall health in mind, promoting a healthier lifestyle alongside OA symptom management as opposed to purely focussing on surgical decision-making.

The primary focus of conversation during these clinical encounters is the potential upcoming surgery. As a result, any discussion around weight loss is often brief, failing to meet the needs of the patient who must leave the consultation motivated to achieve it. A 2019 systematic review of qualitative papers on living with OA highlighted a key theme for this discussion [35]. Patients who had positive interactions felt listened to, hopeful for the future and received information on OA management, including weight loss. Meanwhile, those who reported negative interactions felt dissatisfied at a lack of attention, not being listened to and a lack of information on management options.

In terms of goal setting, BMI has its uses, as discussed above. However, its suitability for routine use in agreeing a tailored weight loss target between surgeon and patient is questionable.

Using a percentage of current body weight provides a promising metric in which surgeons could be agreeing on a personal target with patients. The NICE guidance outlines that overweight or obese OA patients should be advised losing unwanted body weight is beneficial, with a 10% loss better than 5% [34].

A study randomised 86 symptomatic knee OA patients with a BMI ≥30kg/m^2^ to either a weight-stable control group or a weight-loss intervention group [36]. The intervention group were set a goal of 10% loss in initial body weight and were prescribed a large caloric deficit diet alongside 3d/week exercise. Amongst the intervention group (n=43), half of the participants achieved 10% or more weight loss. Only 6 of the 43 failed to reduce their initial body weight by over 5%. The intervention group saw improvements in WOMAC, better performance on the six-minute walk test and quicker stair climb time.

Is it easy to lose weight with knee OA?

A qualitative study by Carmona-Terés et al. looked at understanding patient perspectives on knee OA [37]. Authors describe ‘the impossibility of losing weight’ as being a frequent belief for women with knee OA. Participants also found it difficult to modify behaviours related to losing weight, citing lack of discipline as a primary cause.

In a prospective observational study, 289 morbidly obese patients with advanced knee OA were provided information on risks of Joint replacement in the morbidly obese and given referral details to a bariatric clinic [38]. Only 67 (23.2%) participants attended the bariatric clinic and just 58 (20.1%) underwent a replacement. The median BMI within the sub-set that went on to have surgery was lower than the whole-group median at the initial visit (45.3kg/m^2^ vs. 46.9kg/m^2^). Additionally, BMI within those who received it did reduce from median 45.3kg/m^2^ to 42.3kg/m^2^ between the initial orthopaedic visit and surgery date. Just 23 patients achieved a BMI of < 40kg/m^2^.

This matches the findings of Foreman et al. who retrospectively reviewed moderate or severe hip or knee OA patients with BMI >40kg/m2 plus [39]. They found only 8.9% of patients achieved a BMI <40kg/m^2^ at last follow-up. Additionally, 51.3% of patients above this threshold were absent at follow-up after weight loss counselling and receiving the risks in the morbidly obese. Those who returned for a second visit had relatively lower BMI (46.3kg/m^2^) at the initial visit than the single-visit cohort (49.5kg/m^2^). The participants in this study were given a weight loss goal in pounds based upon their BMI.

Therefore, it could be interpreted that those patients with a BMI further above the 40kg/m^2^ threshold are at greater risk of being lost from care than those relatively lower if a target is set using BMI, with only counselling and education.

Shapiro and colleagues followed 125 patients who were refused a TKR or THR due to a BMI of >40kg/m^2^ [40]. These patients were given a personalised weight loss target based upon their BMI. Only 10.1% of those refused TKR reduced to a 40kg/m^2^ BMI through lifestyle changes.

Howarth et al. conducted a cross-sectional questionnaire amongst obese (BMI >30kg/m^2^) knee OA patients [41]. Of the 45 participants, 89% deemed a lack of motivation as the main weight loss barrier, whilst only 28% saw their knee pain as a barrier to weight loss. The authors concluding remarks re-iterate how important the orthopaedic surgeon is in engaging their patients with a weight loss target.

How should orthopaedic surgeons agree on weight loss goals with patients?

Whilst it is not the role of an orthopaedic surgeon to provide ongoing support with the weight loss journey, they can play an often-pivotal role in education regarding the risks, recognising the scale of the task, showing empathy and then raising a potential course of action which could include signposting to information, onward referral, and recommendation for support from appropriate professionals or groups.

It appears reasonable to conclude that in overweight or obese OA patients, aiming to agree upon a goal of 10% loss in body weight with an initial goal of 5% as a stepping stone is appropriate for orthopaedic surgeons to routinely recommend to overweight/obese OA patients. We must also discuss how best this goal is agreed upon within a short clinic appointment with the aim of making it a positive clinical encounter.

The surgeon should do their utmost to empower their patients to take charge of their weight loss journey. Does this involve more than simply prescribing weight loss? Caneiro and colleagues describe how important it is to ‘put the patient in charge’ of their OA management through building a positive mindset, improving confidence to engage with strengthening and targeting weight loss where needed [42].

There are barriers to healthcare professionals (HCPs) discussing weight loss with patients. In a survey of 14,502 HCPs and 2,785 people with obesity, over half of the HCPs reported limited appointment time as a reason for not discussing weight loss [43]. Importantly, 68% of the people with obesity surveyed appreciated the fact their HCP discussed or would discuss weight loss and only 3% reported feeling offended if their HCP raised weight loss as a topic.

Many HCPs, including surgeons, may avoid discussions about weight loss due to fear it will disrupt the patient-professional relationship [42]. A study in 2011 of overweight or obese patients via semi-structured interview did raise those terms such as ‘obese’, whilst had potential to trigger negative emotion if used without sensitivity, were appropriate for the context of a healthcare appointment [44]. The variation in terminology used when discussing weight loss meant the authors concluded there is not a one-size-fits-all method to weight loss discussion, the clinician must use their understanding to gauge the situation and discuss weight loss in a way they feel the patient will find acceptable. Meanwhile, a cross-sectional study of patients looking for overweight/obesity treatment gathered data on the desirability of particular terms [45]. These participants (n=143) ranked; ‘weight’, ‘excess weight’ and ‘unhealthy body weight’ as the three most usable terms with ‘fatness’, excess fat’ and ‘heaviness’ being the least favourable terms.

Dadabo et al. summarised published literature on weight loss in overweight and obese patients with knee OA [46]. The setting of an individualised, patient-centred weight loss goal is highlighted as crucial and is likely best attained with a combination of dietary changes and physical activity.

Amongst those whose motivation is lower, sustaining a lifestyle of increased physical activity and adhering to a changed diet compared to a previous habitual dietary pattern is highly probable to be a difficult task [3].

Support available to patients to lose weight

Several individuals and agencies are able to support the patient on their journey to losing weight, in addition to new treatment options other than the already discussed. Below are some groups or individuals to which the orthopaedic surgeon could turn to. A good starting point is always the General Practitioner (GP)/ family doctor, who could help in coordination of the different aspects of management. 

Dietitian

A dietitian can help with education and creating a customised meal plan to reduce calorie intake. A referral could be directly to hospital dietitians when local policy allows, or through GP referral.

Physiotherapists

Physiotherapists, preferably one with interest in obesity and OA, can supervise a low-impact exercise program (water exercises, biking etc..) that takes the limitations of knee OA into account.

Local Weight Loss Programs

 Many NHS trusts have their own programs to help patients through group support and education.

NHS online and app support: The NHS Weight Loss Plan is an NHS-supported plan with web and mobile interfaces to help patients lose weight (https://play.google.com/store/apps/details?id=com.nhs.weightloss&hl=en_GB&gl=US&pli=1 for Android and https://apps.apple.com/gb/app/nhs-weight-loss-plan/id1519208548 for IOS). This includes an NHS 12-week weight management program. In July 2020, Public Health England (PHE) launched ‘Better Health’ an adult health campaign encouraging healthier lifestyles. The campaign supports weight management and healthy behaviours amongst people who are overweight or obese, particularly men ≥ 40 years, lower socioeconomic groups, Minority Ethnic groups and those with long-term medical conditions. An updated version of the NHS Weight Loss Plan was launched by PHE as a mobile application. The guides were divided into simple weekly charts of food and exercise, and healthy diet and exercise advice. The mobile application is meant as an interactive and user-friendly version of the plan. A review of the plan on Feb 2021 found that it was downloaded around 865,000 times. Those who completed the plan reduced their original weight by 6.5%, with 64.2% losing more than 5% of their original weight.

Medications

A new class of medications (GLP-1 antagonists) have emerged with good efficacy and low side effects profile for losing weight, which could be considered. The Shanghai Osteoarthritis Cohort study prospectively followed >40 000 adults above 45 years with clinically diagnosed OA and concluded that GLP-1RA in sufficient treatment duration could be knee OA disease-modifying in patients with diabetes, possibly through weight loss [47]. However, they found a need for more investigations to clarify the effects on joint structure, disease process, and patient-reported outcomes.

Bariatric Surgery

This is an option for some patients for whom other methods have failed. A recent literature review suggested that bariatric surgery may improve hip and knee joint pain, but no conclusive evidence exists [48]. In addition, it can reduce the risk of complications after TKR [49]. From a cost-effectiveness point of view, bariatric surgery offers good value for money for those patients [50].

Psychological Support

Counselling can address underlying issues driving or resulting from obesity or OA.

## Conclusions

Orthopaedic surgeons should be confident when recommending weight loss to overweight/obese OA patients, recognise the potential difficulty of the task to that individual and approach the conversation in a way judged to be appropriate given that context. The primary aim of this discussion should be for patients to leave motivated to begin a weight loss journey, have some actionable advice to begin self-management and have a goal that has been agreed with their surgeon, with direction to the appropriate support group helping along this journey.

## References

[1] Leifer VP, Katz JN, Losina E (2022). The burden of OA-health services and economics. Osteoarthritis Cartilage.

[2] Hunter DJ, Bierma-Zeinstra S (2019). Osteoarthritis. Lancet.

[3] Bliddal H, Leeds AR, Christensen R (2014). Osteoarthritis, obesity and weight loss: evidence, hypotheses and horizons - a scoping review. Obes Rev.

[4] Salis Z, Sainsbury A (2023). Association between change in body mass index and knee and hip replacements: a survival analysis of seven to ten years using multicohort data. Arthritis Care Res (Hoboken).

[5] Maston G, Kahlaee HR, Franklin J (2022). Real world adherence to a severely energy restricted meal replacement diet in participants with Class II and III obesity. Obesities.

[6] Maston G, Franklin J, Hocking S (2021). Dietary adherence and program attrition during a severely energy-restricted diet among people with complex class III obesity: a qualitative exploration. PLoS One.

[7] Jin X, Gibson AA, Gale J (2021). Does weight loss reduce the incidence of total knee and hip replacement for osteoarthritis?-a prospective cohort study among middle-aged and older adults with overweight or obesity. Int J Obes (Lond).

[8] Christensen R, Bartels EM, Astrup A, Bliddal H (2007). Effect of weight reduction in obese patients diagnosed with knee osteoarthritis: a systematic review and meta-analysis. Ann Rheum Dis.

[9] Salis Z, Sainsbury A, I Keen H, Gallego B, Jin X (2022). Weight loss is associated with reduced risk of knee and hip replacement: a survival analysis using Osteoarthritis Initiative data. Int J Obes (Lond).

[10] Joseph GB, McCulloch CE, Nevitt MC, Lynch J, Lane NE, Link TM (2023). Effects of weight change on knee and hip radiographic measurements and pain over four years: data from the Osteoarthritis Initiative. Arthritis Care Res (Hoboken).

[11] Reyes C, Leyland KM, Peat G, Cooper C, Arden NK, Prieto-Alhambra D (2016). Association between overweight and obesity and risk of clinically diagnosed knee, hip, and hand osteoarthritis: a population-based cohort study. Arthritis Rheumatol.

[12] Jiang L, Tian W, Wang Y (2012). Body mass index and susceptibility to knee osteoarthritis: a systematic review and meta-analysis. Joint Bone Spine.

[13] Messier SP, Resnik AE, Beavers DP (2018). Intentional weight loss in overweight and obese patients with knee osteoarthritis: is more better?. Arthritis Care Res (Hoboken).

[14] Riddle DL, Stratford PW (2013). Body weight changes and corresponding changes in pain and function in persons with symptomatic knee osteoarthritis: a cohort study. Arthritis Care Res (Hoboken).

[15] Giori NJ, Amanatullah DF, Gupta S, Bowe T, Harris AH (2018). Risk reduction compared with access to care: quantifying the trade-off of enforcing a body mass index eligibility criterion for joint replacement. J Bone Joint Surg Am.

[16] Mishra AK, Vaish A, Vaishya R (2022). Effect of body mass index on the outcomes of primary total knee arthroplasty up to one year - a prospective study. J Clin Orthop Trauma.

[17] Chen JY, Xu S, Pang HN, Tay DK, Chia SL, Lo NN, Yeo SJ (2018). Change in body mass index after total knee arthroplasty and its influence on functional outcome. J Arthroplasty.

[18] Wunderlich F, Eckhard L, Büttner M (2023). The INDICATE Knee expectations survey detects general patient treatment goals for total knee arthroplasty and the influence of demographic factors on patients expectations. Knee Surg Sports Traumatol Arthrosc.

[19] Polat AE, Polat B, Gürpınar T, Çarkçı E, Güler O (2019). The effect of morbid obesity (BMI ≥ 35 kg/m(2)) on functional outcome and complication rate following unicompartmental knee arthroplasty: a case-control study. J Orthop Surg Res.

[20] Ponnusamy KE, Marsh JD, Somerville LE, McCalden RW, Vasarhelyi EM (2018). Ninety-day costs, reoperations, and readmissions for primary total knee arthroplasty patients with varying body mass index levels. J Arthroplasty.

[21] Seward MW, Briggs LG, Bain PA, Chen AF (2021). Preoperative nonsurgical weight loss interventions before total hip and knee arthroplasty: a systematic review. J Arthroplasty.

[22] Arnold N, Anis H, Barsoum WK (2020). Preoperative cut-off values for body mass index deny patients clinically significant improvements in patient-reported outcomes after total hip arthroplasty. Bone Joint J.

[23] Werner BC, Higgins MD, Pehlivan HC, Carothers JT, Browne JA (2017). Super obesity is an independent risk factor for complications after primary total hip arthroplasty. J Arthroplasty.

[24] DeMik DE, Bedard NA, Dowdle SB, Elkins JM, Brown TS, Gao Y, Callaghan JJ (2018). Complications and obesity in arthroplasty-a hip is not a knee. J Arthroplasty.

[25] Middleton FR, Boardman DR (2007). Total hip arthroplasty does not aid weight loss. Ann R Coll Surg Engl.

[26] Ast MP, Abdel MP, Lee YY, Lyman S, Ruel AV, Westrich GH (2015). Weight changes after total hip or knee arthroplasty: prevalence, predictors, and effects on outcomes. J Bone Joint Surg Am.

[27] Li W, Ayers DC, Lewis CG, Bowen TR, Allison JJ, Franklin PD (2017). Functional gain and pain relief after total joint replacement according to obesity status. J Bone Joint Surg Am.

[28] Judge A, Batra RN, Thomas GE (2014). Body mass index is not a clinically meaningful predictor of patient reported outcomes of primary hip replacement surgery: prospective cohort study. Osteoarthritis Cartilage.

[29] Messier SP, Mihalko SL, Legault C (2013). Effects of intensive diet and exercise on knee joint loads, inflammation, and clinical outcomes among overweight and obese adults with knee osteoarthritis: the IDEA randomized clinical trial. JAMA.

[30] Peeler J, Ripat J (2018). The effect of low-load exercise on joint pain, function, and activities of daily living in patients with knee osteoarthritis. Knee.

[31] Avery A, Langley-Evans SC, Harrington M, Swift JA (2016). Setting targets leads to greater long-term weight losses and 'unrealistic' targets increase the effect in a large community-based commercial weight management group. J Hum Nutr Diet.

[32] Arden NK, Perry TA, Bannuru RR (2021). Non-surgical management of knee osteoarthritis: comparison of ESCEO and OARSI 2019 guidelines. Nat Rev Rheumatol.

[33] Katz JN, Arant KR, Loeser RF (2021). Diagnosis and treatment of hip and knee osteoarthritis: a review. JAMA.

[34] (2022). NICE Guidelines. Online.

[35] Wallis JA, Taylor NF, Bunzli S, Shields N (2019). Experience of living with knee osteoarthritis: a systematic review of qualitative studies. BMJ Open.

[36] Miller GD, Nicklas BJ, Davis C, Loeser RF, Lenchik L, Messier SP (2006). Intensive weight loss program improves physical function in older obese adults with knee osteoarthritis. Obesity (Silver Spring).

[37] Carmona-Terés V, Moix-Queraltó J, Pujol-Ribera E (2017). Understanding knee osteoarthritis from the patients' perspective: a qualitative study. BMC Musculoskelet Disord.

[38] Springer BD, Roberts KM, Bossi KL, Odum SM, Voellinger DC (2019). What are the implications of withholding total joint arthroplasty in the morbidly obese? A prospective, observational study. Bone Joint J.

[39] Foreman CW, Callaghan JJ, Brown TS, Elkins JM, Otero JE (2020). Total joint arthroplasty in the morbidly obese: how body mass index ≥40 influences patient retention, treatment decisions, and treatment outcomes. J Arthroplasty.

[40] Shapiro JA, Narayanan AS, Taylor PR, Olcott CW, Del Gaizo DJ (2020). Fate of the morbidly obese patient who is denied total joint arthroplasty. J Arthroplasty.

[41] Howarth D, Inman D, Lingard E, McCaskie A, Gerrand C (2010). Barriers to weight loss in obese patients with knee osteoarthritis. Ann R Coll Surg Engl.

[42] Caneiro JP, O'Sullivan PB, Roos EM (2020). Three steps to changing the narrative about knee osteoarthritis care: a call to action. Br J Sports Med.

[43] Caterson ID, Alfadda AA, Auerbach P (2019). Gaps to bridge: misalignment between perception, reality and actions in obesity. Diabetes Obes Metab.

[44] Gray CM, Hunt K, Lorimer K, Anderson AS, Benzeval M, Wyke S (2011). Words matter: a qualitative investigation of which weight status terms are acceptable and motivate weight loss when used by health professionals. BMC Public Health.

[45] Dutton GR, Tan F, Perri MG, Stine CC, Dancer-Brown M, Goble M, Van Vessem N (2010). What words should we use when discussing excess weight?. J Am Board Fam Med.

[46] Dadabo J, Fram J, Jayabalan P (2019). Noninterventional therapies for the management of knee osteoarthritis. J Knee Surg.

[47] Zhu H, Zhou L, Wang Q (2023). Glucagon-like peptide-1 receptor agonists as a disease-modifying therapy for knee osteoarthritis mediated by weight loss: findings from the Shanghai Osteoarthritis Cohort. Ann Rheum Dis.

[48] Heuts EA, de Jong LD, Hazebroek EJ, Wagener M, Somford MP (2021). The influence of bariatric surgery on hip and knee joint pain: a systematic review. Surg Obes Relat Dis.

[49] Dowsey MM, Brown WA, Cochrane A, Burton PR, Liew D, Choong PF (2022). Effect of bariatric surgery on risk of complications after total knee arthroplasty: a randomized clinical trial. JAMA Netw Open.

[50] Kostic AM, Leifer VP, Gong Y (2023). Cost-effectiveness of surgical weight-loss interventions for patients with knee osteoarthritis and Class III obesity. Arthritis Care Res (Hoboken).

